# Musashi-1 promotes cancer stem cell properties of glioblastoma cells via upregulation of YTHDF1

**DOI:** 10.1186/s12935-020-01696-9

**Published:** 2020-12-14

**Authors:** Aliaksandr A. Yarmishyn, Yi-Ping Yang, Kai-Hsi Lu, Yi-Chen Chen, Yueh Chien, Shih-Jie Chou, Ping-Hsing Tsai, Hsin-I. Ma, Chian-Shiu Chien, Ming-Teh Chen, Mong-Lien Wang

**Affiliations:** 1 Division of Basic Research, Department of Medical Research, Taipei Veterans General Hospital, 112 Taipei, Taiwan; 2 School of Medicine, National Yang-Ming University, 112 Taipei, Taiwan; 3 School of Pharmaceutical Sciences, National Yang-Ming University, 112 Taipei, Taiwan; 4 Department of Medical Research and Education, Cheng-Hsin General Hospital, 112 Taipei, Taiwan; 5 Department of Neurological Surgery, Tri-Service General Hospital and National Defense Medical Center, 114 Taipei, Taiwan; 6 Institute of Pharmacology, National Yang-Ming University, 112 Taipei, Taiwan; 7 Department of Neurosurgery, Taipei Veterans General Hospital, 112 Taipei, Taiwan; 8 Institute of Food Safety and Health Risk Assessment, National Yang Ming University, 112 Taipei, Taiwan

**Keywords:** YTHDF1, Musashi-1, Glioblastoma, Cancer progression

## Abstract

**Background:**

Glioblastoma (GBM) is the most lethal brain tumor characterized by high morbidity and limited treatment options. Tumor malignancy is usually associated with the epigenetic marks, which coordinate gene expression to ascertain relevant phenotypes. One of such marks is m6A modification of RNA, whose functional effects are dependent on the YTH family m6A reader proteins.

**Methods and results:**

In this study, we investigated the expression of five YTH family proteins in different GBM microarray datasets from the Oncomine database, and identified YTHDF1 as the most highly overexpressed member of this family in GBM. By performing the knockdown of YTHDF1 in a GBM cell line, we found that it positively regulates proliferation, chemoresistance and cancer stem cell-like properties. Musashi-1 (MSI1) is a postranscriptional gene expression regulator associated with high oncogenicity in GBM. By knocking down and overexpressing MSI1, we found that it positively regulates YTHDF1 expression. The inhibitory effects imposed on the processes of proliferation and migration by YTHDF1 knockdown were shown to be partially rescued by concomitant overexpression of MSI1. MSI1 and YTHDF1 were shown to be positively correlated in clinical glioma samples, and their concomitant upregulation was associated with decreased survival of glioma patients. We identified the direct regulation of YTHDF1 by MSI1.

**Conclusions:**

Given the fact that both proteins are master regulators of gene expression, and both of them are unfavorable factors in GBM, we suggest that in any future studies aimed to uncover the prognostic value and therapy potential, these two proteins should be considered together.

## Background

Gliomas represent the most common type of primary brain tumors originating from glial cells. Glioblastoma (GBM) is the most fatal type of glioma classified by the World Health Organization as a grade IV tumor [1]. GBM is characterized by high level of heterogeneity and pleomorphic morphology, highly infiltrative nature, which allows rapid spread into neighboring brain tissues. The conventional treatment protocol includes maximal safe surgical resection followed by radiotherapy and concomitant chemotherapy with an alkylating agent temozolomide (TMZ), however, the prognosis even for the patients receiving treatment remains dismal with the median survival of only about 14.6 months [2].

Nowadays, it became increasingly clear that tumors are composed of a heterogeneous population of cells that includes a subpopulation of self-renewing cells with stem cell properties, known as cancer stem cells (CSCs). CSCs are able to initiate tumor growth, and are more resistant to chemotherapy treatment, which makes them an important factor for tumor relapse. GBM is characterized by high degree of cellular, genetic and epigenetic heterogeneity, and the presence of CSCs is believed to be a major determinant for tumor therapy resistance, recurrence and invasive growth [3, 4].

Musashi-1 (MSI1) is a highly conserved RNA-binding protein (RBP) that is overexpressed in GBM and serves as an unfavorable prognostic biomarker [5, 6]. In vertebrates, MSI1 has been initially identified to be overexpressed in neuronal stem cells within the CNS, but not in the differentiated neurons or glial cells [7]. Further studies have confirmed the role of MSI1 in the maintenance of stem cells in various tissues [8]. Consistently with its high expression in cancers, and its importance in stem cell signaling, MSI1 has been implicated in CSC properties of different tumors, including GBM [9, 10]. The most well-characterized mode of action of MSI1 as an RBP is by inhibiting the translation of target mRNAs. For example, binding to 3′-UTR of *NUMB* mRNA, encoding a repressor of the Notch signaling pathway, results in inhibition of its translation, which leads to derepression of Notch pathway required for the maintenance of stemness [11]. However, in different cellular contexts, MSI can also act as an activator of translation [12].

Nowadays, it is widely accepted that transitions between different cellular states, such as between pluripotency and differentiation, are associated with the global-scale changes in the epigenome. Recent evidence indicates that epitranscriptomic networks may play equally important roles in affecting the balance between pluripotent and differentiated states, and therefore, may have an impact on CSC properties of tumors [13, 14]. N6-methyl-adenosine (m6A) is the most prevalent mRNA modification, which has recently been shown to play an important role in cell fate transitions [13]. Whereas m6A marks are imposed and erased by the methyltransferases (m6A writers) and demethylases (m6A erasers), respectively, a group of RBPs of the YTH domain family, known as m6A readers, is responsible for the functional effects of m6A modifications of mRNA. m6A readers include five members of the YTH family of proteins, YTHDF1, YTHDF2, YTHDF3, YTHDC1, and YTHDC2, which recruit m6A-tagged mRNA into different pathways of RNA metabolism [15]. Nuclear-localized YTHDC1 regulates alternative splicing [16], YTHDF1 and YTHDF3 promote mRNA translation [17, 18], whereas YTHDF2 destabilizes m6A-tagged mRNA [19].

In this study, we aimed to find a link between MSI1 and m6A-mediated epitranscriptomic pathways in regulating the malignancy of GBM. We identified YTHDF1 as the most highly overexpressed m6A reader protein in GBM, and found it to be directly involved in regulating the proliferation of a GBM cell line, as well increasing its resistance to TMZ, and augmenting the CSC characteristics. We found that YTHDF1 is positively regulated by MSI1, and YTHDF1 mediates the effect of MSI1 on GBM cell proliferation and migration capacity.

## Methods

### Cell culture

The human GBM cell line DBTRG-05MG was obtained from the American Type Culture Collection (ATCC) before 2007 and tested positive for human origin. DBTRG-05MG cell line was cultured in Dulbecco’s Modified Eagle’s Media (DMEM; Thermo Fisher Scientific, Waltham, MA, USA) supplemented with 10% fetal bovine serum (FBS; GE Healthcare, Chicago, IL, USA), 150 g/mL G418 (Sigma-Aldrich, St. Louis, MO, USA), 100 units/mL penicillin, and 100 µg/mL streptomycin (Thermo Fisher Scientific) under standard culture condition (37 °C, 95% humidified air and 5% CO2). Subculturing was performed using 0.25% trypsin-EDTA (Sigma-Aldrich). Cells were tested for mycoplasma contamination.

### Transduction of lentivirus shRNA-coding vectors

The day before transduction, Platinum-A cells were seeded in a 10-cm dish. Next day, either pLKO.1 base lentiviral vector or pLKO.1-shYTHDF1 construct were introduced into Platinum-A cells using TransIT-LT1 transfection reagent (Mirus Bio, Madison, WI, USA). 24 h after transfection, the medium was replaced with normal culture medium. After 24 h, virus-containing supernatant derived from these Platinum-A cultures was filtered through 0.45 µm cellulose acetate filter (Pall Corporation, Port Washington, NY, USA) and supplemented with 8 µg/ml Polybrene (Sigma-Aldrich). Target DBTRG-05MG cells were incubated in the virus/Polybrene-containing supernatants for 24 h. On the next day, the supernatant was replaced with fresh medium.

### Plasmid transfection

MSI1 coding sequence was amplified from human cDNA using primers introducing HindIII and BamH1 restriction sites. The FLAG-tagged MSI1-encoding plasmid was generated by inserting a 1038-bp fragment of full-length human MSI1 cDNA into the HindIII/BamHI site of p3xFLAG-Myc-CMV-26 vector (Sigma-Aldrich). In vitro plasmid transfection was carried out using Lipofectamine 2000 (Thermo Fisher Scientific) according to the manufacturer’s instructions.

### Gene silencing with siRNA

siRNAs against MSI1 (Cat. No. SASI_Hs01_00145278), YTHDF1 (#1 Cat. No.: SASI_Hs01_00233686; #2 Cat. No.: SASI_Hs01_00233687), and scrambled control (Cat. No. SAS-SIRDU10D) used in the knockdown experiments were purchased from Sigma-Aldrich. shRNAs against YTHDF1 were purchased from the RNAiCore of Academia Sinica, Taiwan (Cat. No. TRCN0000062771 and TRCN0000294275). Transient transfection of siRNA was carried out at a 50 nM final concentration with Lipofectamine RNAiMAX (Thermo Fisher Scientific) according to the manufacturer’s protocol. RNA and cell-based experiments were performed after 48 h of incubation. Stable transfection of shRNAs was carried out using Lipofectamine 2000 (Thermo Fisher Scientific) according to the manufacturer’s instruction.

### Western blotting

Cells were lysed with RIPA buffer (Thermo Fisher Scientific) containing 1% protease inhibitor, and non-soluble cell debris were removed by centrifugation at 13,200 rpm at 4 °C for 20 min. The supernatant total lysates were transferred to a new tube and protein concentration was determined by the Bradford method (Bio-Rad Protein Assay). Equal weights of total protein were separated by electrophoresis on SDS/PAGE. After the proteins had been transferred onto a polyvinylidene difluoride (PVDF) membrane (Millipore, Bedford, MA, USA), the blots were incubated with blocking buffer (1 X PBST and 5% skim milk) for 1 h at room temperature and then hybridized with primary antibodies overnight at 4 °C, followed by incubation with horseradish peroxidase-conjugated secondary antibody for 1 h at room temperature. The blots were obtained by X-ray film exposure, and the intensities were quantified by densitometry analysis (Digital Protein DNA Imagineware, Huntington Station, NY, USA). The following primary antibodies were used: rabbit monoclonal anti-Musashi-1 (#5663; Cell Signaling Technology, Danvers, MA, USA), rabbit monoclonal anti-Musashi-1 (ab52865; Abcam, Cambridge, UK), mouse monoclonal anti-FLAG M2 (F1804; Sigma-Aldrich), rabbit polyclonal anti-YTHDF1 (17479-1-AP; Proteintech Group, Chicago, IL, USA), rabbit polyclonal anti-YTHDF2 (24744-1-AP; Proteintech Group), rabbit polyclonal anti-YTHDF3 (25537-1-AP; Proteintech Group), rabbit polyclonal anti-SOX2 (#2748; Cell Signaling Technology), rabbit monoclonal anti-NANOG (#4903; Cell Signaling Technology), mouse monoclonal anti-CD133 (14-1331-82; Thermo Fisher Scientific), mouse monoclonal anti-GAPDH (a8795; Sigma-Aldrich). The following secondary antibodies were used: anti-mouse IgG HRP-linked (#7076, Cell Signaling Technology), anti-rabbit IgG HRP-linked (#7074, Cell Signaling Technology).

### RNA extraction and quantitative real-time PCR (qRT-PCR)

Total RNA was extracted using TRIzol reagent (Thermo Fisher Scientific) according to the manufacturer’s protocol. Total RNA was used as a template for SuperScript III Reverse Transcriptase (Thermo Fisher Scientific) to obtain single-stranded cDNA. Quantitative real-time PCR (qRT-PCR) was performed with Power SYBR Green Master Mix (Thermo Fisher Scientific) according to the manufacturer’s instructions. Oligonucleotide specificity was tested by BLAST (National Center for Biotechnology Information, Bethesda, MD, USA) homology search with the human genome and later confirmed by melting curve analysis. The following pairs of primers were used to amplify the respective transcripts: YTHDF1 (forward TCCTACAAGCACACAACCTCCA, reverse TTTCGACTCTGCCGTTCCTT), MSI1 (forward TTGACAAAACCACCAACCGG, reverse CCTCCTTTGGCTGAGCTTTCTT), 18S (forward GGCGGCGTTATTCCCATGA, reverse GAGGTTTCCCGTGTTGAG). Signals were detected on an 7900HT Fast Real-Time PCR System (Thermo Fisher Scientific).

### Sphere-formation assay

Cells were seeded at the density of 2,000 cells/well in 24-well plates in serum-free DMEM:F12 medium supplemented with N-2 supplement (Thermo Fisher Scientific), heparin (4 µg/ml), fresh human EGF (20 ng/ml) and bFGF (20 ng/ml) (PeproTech, Rocky Hill, NJ, USA). Cells were incubated at 37 °C in a humidified 5% CO2 atmosphere, and the fresh culture medium was added once a week until cells started to form floating aggregates. Two weeks after seeding, the surface area of spheres was measured using ImageJ software.

### MTT in vitro proliferation assay

Cells were seeded at a density of 10,000 cells/well in a 12-well dish and allowed to grow at 37 °C with 5% CO2. On the following day, cells were transfected with scrambled control or YTHDF1-specific siRNA. Growth was assayed for 3 days; every day 500 µl/well of MTT reagent (0.5 mg/ml) was added and incubation was carried out at 37 °C with 5% CO2 for 1 h. The medium was aspirated, and 500 µl of DMSO was added and mixed until purple color was formed. 200 µl of the cell samples were measured using a plate reader at 560 nm and 670 nm. Growth curves were constructed according to the collected data.

### TMZ chemoresistance assay

Cells were seeded into 12-well dishes at a density of 15,000 cells/well with complete growth medium. TMZ was added at different concentrations (0.5, 2, and 3 mM) and DMSO (solvent) was added to the control batch of cells. Cell viability was assessed by MTT assay. In brief, cell were stained with 0.1 mg/ml 3-(4,5-cimethylthiazol-2-yl)-2,5-diphenyl tetrazolium bromide (MTT, Sigma-Aldrich) for 2 h and the formazan crystals were then dissolved in DMSO. The relative absorbance was then measured by TECAN Sunrise microplate absorbance reader (Thermo Fisher Scientific) at 570 nm light absorbance.

### Transwell migration assay

The cell migration assay was performed using FluoroBlok 24-Multiwell Insert System with 8-mm pore size polyethylene terephthalate membrane (Corning Inc., Corning, NY, USA). Briefly, at 48 h post-transfection, 1.5 × 104 cells in 200 µl medium were added to the upper chamber. The lower chamber was filled with 0.7 ml culture medium. Cells were then incubated for 24 h at 37 °C. Cells that migrated to the bottom of the membrane were fixed with pre-chilled methanol at room temperature for 30 min, and stained with 50 µg/ml propidium iodide (Sigma-Aldrich) for 30 min. Finally, stained cells were counted under an inverted fluorescent microscope. To minimize the bias rate, at least three randomly selected fields with 100× magnification were analyzed, and the average number was taken.

### Wound healing cell migration assay

For wound healing cell migration assay, 2 × 10^5^ cells were seeding into each silicon culture insert (ibidi GmbH, Planegg, Germany) in a 24-well cell culture plate and allowed to adhere overnight. Silicon inserts were removed and cells were washed with PBS twice. Each well of the 24-well plate was filled with 1 ml of culture medium or the mixture of DMEM culture medium with conditioned medium (1:1), and the migratory cells were imaged with an inverted microscope. Wound area recovery by migrated cells was quantified by Image J software.

### Measurement of mRNA half-life

DBTRG-05MG cells were seeded at a density of 250,000 cells per 60-mm dish and allowed to attach overnight. Cells were transfected with scrambled control or YTHDF1- specific siRNA using Lipofectamine RNAiMAX (Thermo Fisher Scientific). 24 h later, cells were treated with 5 µg/ml actinomycin D (Sigma-Aldrich), and cells were lysed for total RNA collection at the indicated time points.

### Statistical analysis

Quantitative data are expressed as the mean ± SD from at least three independent experiments. The comparison between groups was performed using Student’s t-test. Differences were considered significant when p ≤ 0.01 (*p ≤ 0.05; **p ≤ 0.01; ***p ≤ 0.005). The data for Kaplan-Meier survival analysis were downloaded from The Cancer Genome Atlas (TCGA) database. In total, 667 samples were analyzed. The original expression data were normalized by fragments per kilobase of transcript per million mapped reads upper quartile (FPKM-UQ) and high and low expression were defined as above the upper quartile and blow the lower quartile, respectively.

## Results

### ***YTHDF1*** mRNA is upregulated in GBM samples compared with normal brain samples in the Oncomine database

**Fig. 1:**
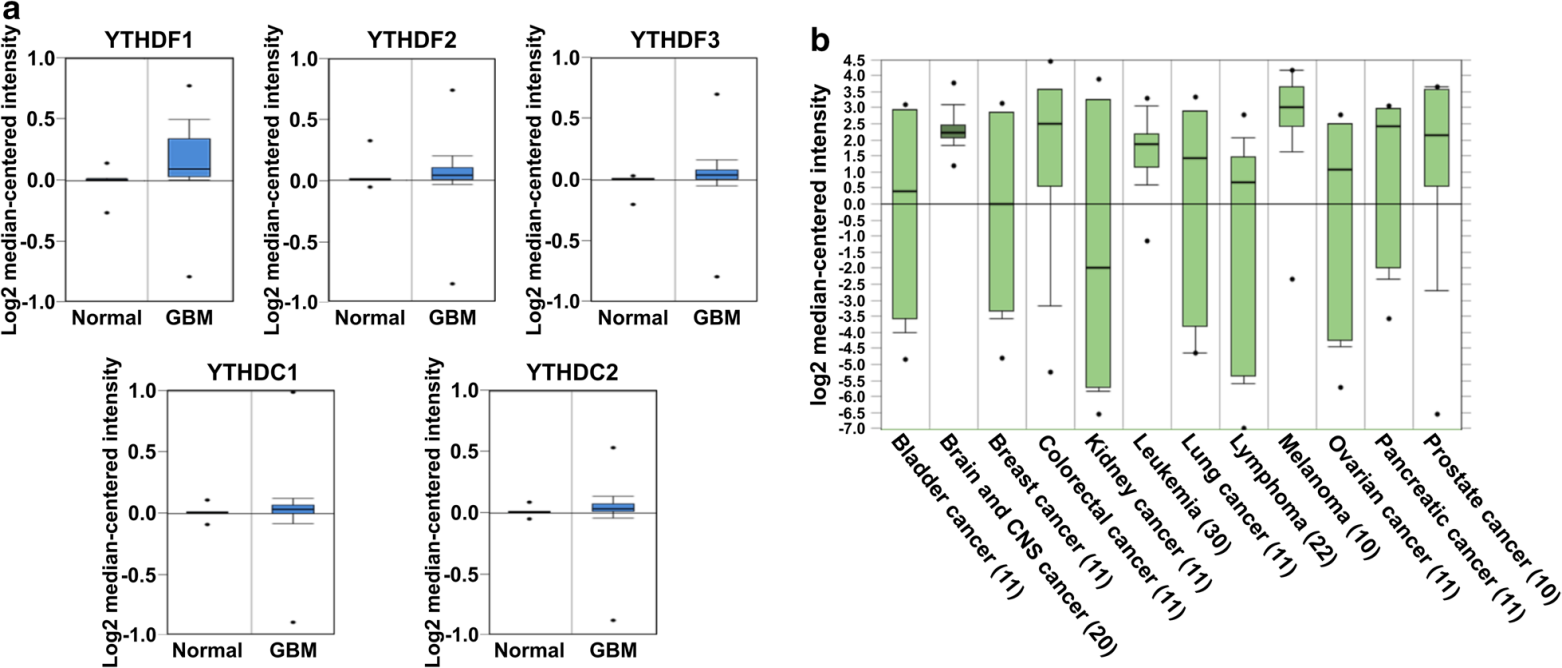
YTHDF1 mRNA is upregulated in GBM samples compared with normal brain samples in the Oncomine database. **a** Analysis of the expression levels of the YTH domain family members in microarray datasets of normal brain tissue (N = 720) and GBM (N = 582) retrieved from the Oncomine database. **b** Analysis of the expression levels of YTHDF1 in different types of cancer in datasets retrieved from the Oncomine database. The numbers of datasets with statistically significant mRNA levels are shown. The threshold parameters were the following: P-value < 1E−4, fold change > 2, and 10% top ranked genes were analyzed (P value = 3.42E-−11)

The YTH family of m6A reader proteins are the key regulators that functionalize the specific m6A modification of mRNA transcripts by modulating their translation and stability. The dysregulation of the expression of YTH family proteins, such as YTHDF1 and YTHDF2, has been shown to promote lung and liver carcinogenesis, respectively [20–22]. The involvement of m6A readers in brain tumor progression, however, has not yet been clearly investigated. Therefore, to identify the potential candidate m6A reader proteins that may play a role in GBM, we utilized the Oncomine database to evaluate the expression levels of m6A readers in normal and GBM samples. We used the TCGA microarray database and compared the mRNA expression levels of five YTH domain family proteins, YTHDF1, YTHDF2, YTHDF3, YTHDC1, and YTHDC2, between 720 normal brain tissue microarray datasets and 582 glioblastoma datasets. Among these five YTH family members, YTHDF1 was found to be the most highly upregulated in GBM samples compared with normal brain samples (Fig. 1a). Next, we also analyzed the expression of YTHDF1 in different types of cancer by analyzing the Ramaswamy multi-cancer dataset from the Oncomine database (Fig. 1b) [23, 24]. We found that YTHDF1 was predominantly upregulated in brain and CNS cancer (n = 20), leukemia (n = 30), and melanoma (n = 10) datasets (Fig. 1b). Taken together, our analysis on the online public database reveal a potential significant involvement of YTHDF1 in brain and CNS cancer.

### Knockdown of YTHDF1 inhibits proliferation and sensitizes GBM cells to TMZ

**Fig. 2:**
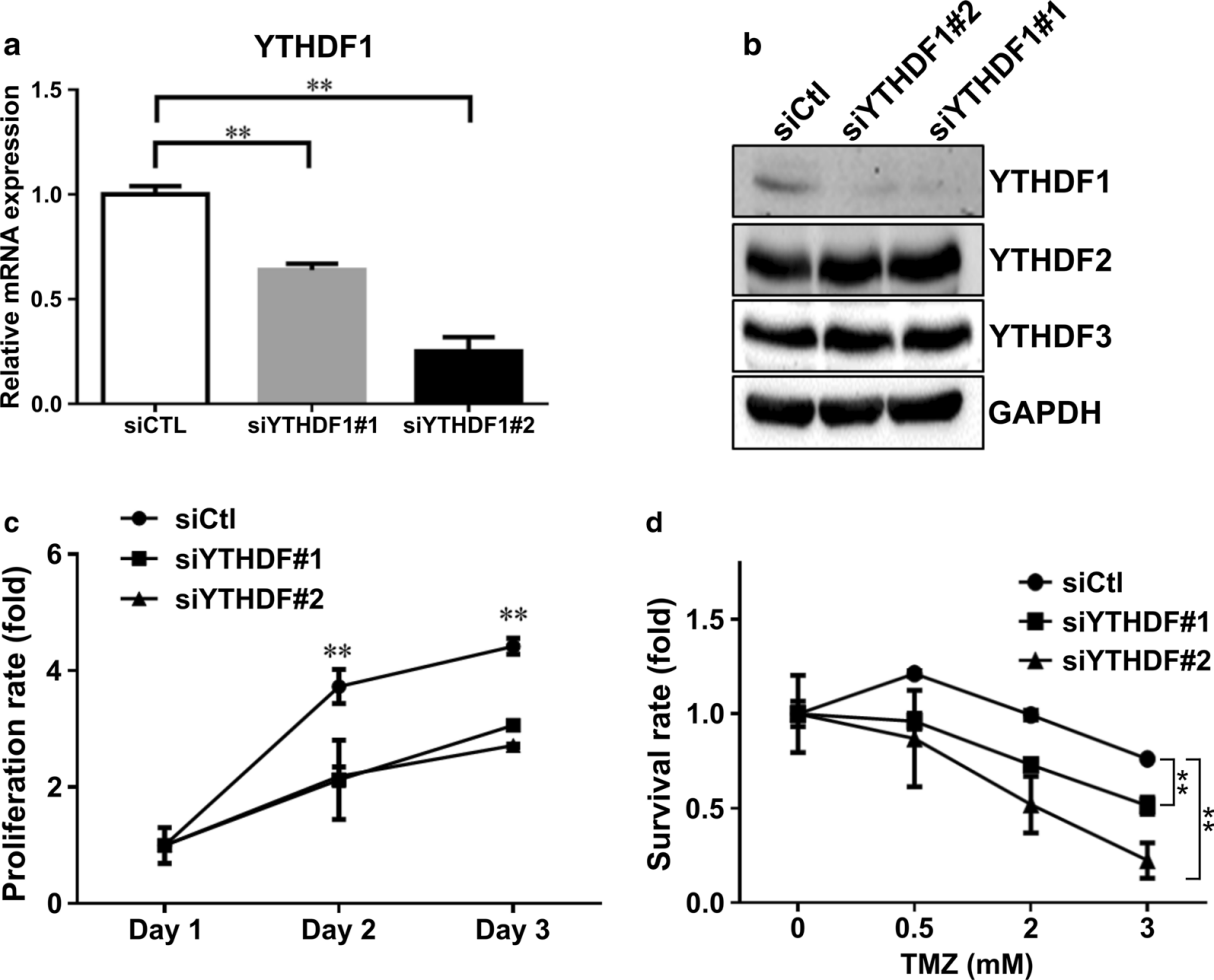
Knockdown of YTHDF1 inhibits proliferation and sensitizes GBM cells to TMZ.** a** qRT-PCR showing the expression levels of *YTHDF1* mRNA in DBTRG-05MG cells transfected with two siRNAs (siYTHDF1#1 and siYTHDF1#2). Data are expressed relative to cells transfected with control siRNA (siCtl). Means from three separate experiments are shown with SD error bars. **P < 0.01 vs. siCtl (Student’s t-test). **b** Western blotting showing protein expression levels of YTHDF1 in DBTRG-05MG cells with YTHDF1 knockdown by two siRNAs (siYTHDF1#1 and siYTHDF1#2) and control (siCtl). YTHDF2 and YTHDF2 are shown as a control for siRNA specificity, GAPDH was used as a loading control. **c** Cell proliferation determined by MTT assay performed on control (siCtl) and YTHDF1-silenced (siYTHDF#1 and siYTHDF1#2) DBTRG-05MG cells. The proliferation rate was calculated as the mean ratio of MTT absorbance on days 2 and 3 to that on day 1. Means from three separate experiments are shown with SD error bars. **P < 0.01 comparing siYTHDF1 vs. siCtl (Student’s t-test). **d** Cell viability determined by MTT assay performed on control (siCtl) and YTHDF1-silenced (siYTHDF#1 and siYTHDF1#2) DBTRG-05MG cells treated with the indicated concentrations of TMZ for 16 h. Data expressed as mean ratios of MTT absorbances at 0.5 mM, 2 mM, and 3 mM TMZ to that at 0 mM TMZ. Three independent experiments were performed and SD error bars are shown, **P < 0.01 (Student’s t-test)

Following the identification of the potential involvement of YTHDF1 in brain tumor, we sought to assess the biological effect of *YTHDF1* knockdown on GBM cells. DBTRG-05MG GBM-derived cells were transfected with two siRNAs, siYTHDF1#1 and siYTHDF1#2, which knocked down *YTHDF1* expression by 50% and 75%, respectively, as was demonstrated by qRT-PCR (Fig. 2a) and western blotting (Fig. 2b). DBTRG-05MG cells showed significantly reduced proliferation rate upon *YTHDF1* knockdown as was determined by MTT assay (Fig. 2c). Temozolomide (TMZ), which is the major drug for GBM chemotherapy, reduced the viability of DBTRG-05MG cells in a concentration-dependent manner (Fig. 2d). Notably, knockdown of *YTHDF1* significantly augmented TMZ cytotoxic effect on GBM cells, which was more pronounced in cells transfected with more efficient siYTHDF1#2 (Fig. 2d). To conclude, YTHDF1 expression in GBM cells is required for proliferation and TMZ drug resistance.

### YTHDF1 is required for maintaining cancer stem cell properties of GBM cell line

**Fig. 3:**
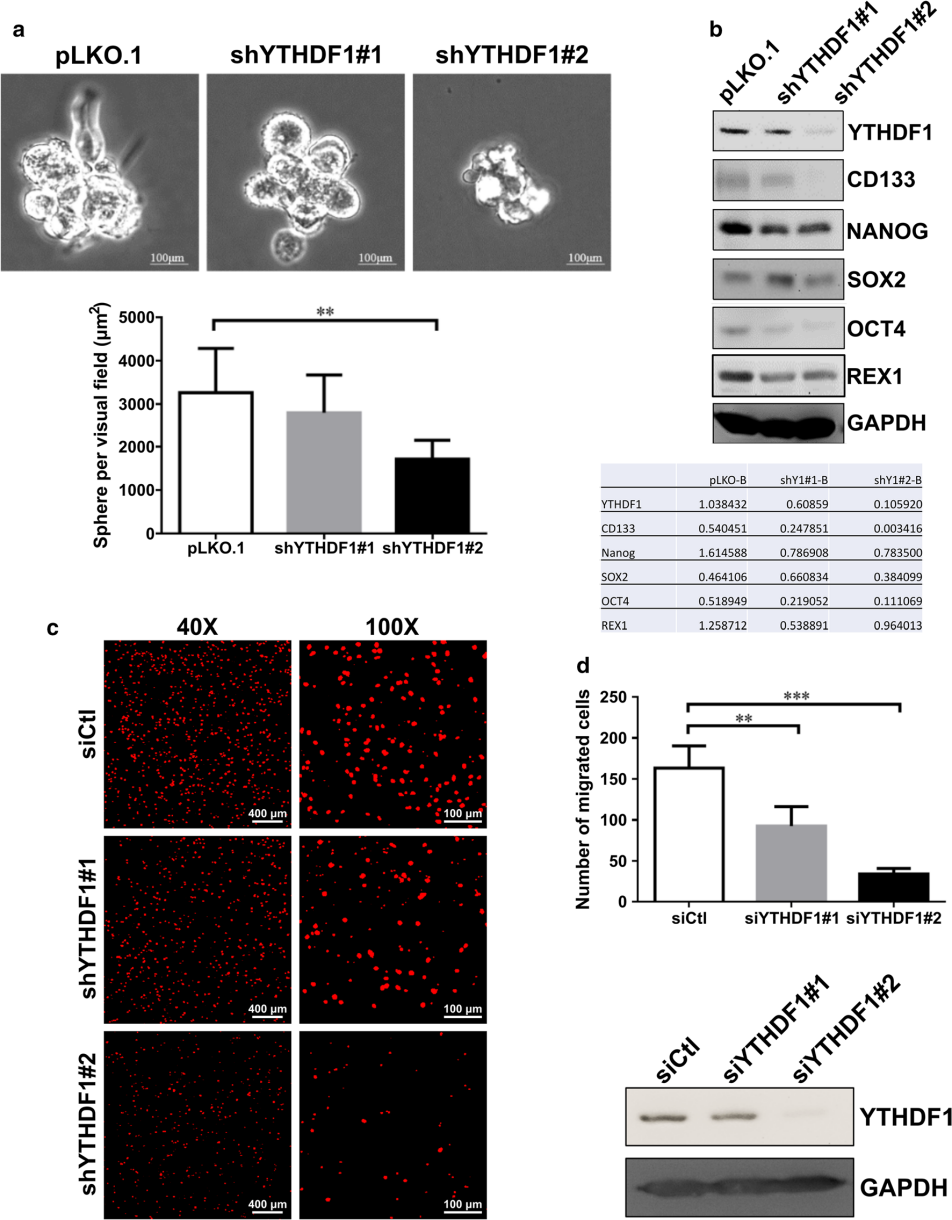
YTHDF1 is required for maintaining cancer stem cell properties of GBM cell line.** a** Sphere- formation assay performed on DBTRG-05MG cells transduced with control (pLKO.1) and two YTHDF1-targeting shRNA constructs (shYTHDF#1 and shYTHDF1#2). Top panel: the first generation spheres formed on day 7. Bottom panel: quantification of sphere area. The values represent the means ± SD error bars from three independent experiments. **P < 0.01 (Student’s t-test) vs. pLKO.1. **b** Western blotting analysis of expression of YTHDF1 and stemness markers, CD133, NANOG, SOX2, OCT4 and REX1, in sphere cells. GAPDH used as a loading control. **c** Transwell migration assay performed on DBTRG-05MG cells transfected with control (siCtl) and YTHDF-targeting siRNAs (siYTHDF1#1 and siYTHDF1#2). Cells that migrated from the top to the underside of transwell filters in 24 h were fixed and stained with PI. **d** Top panel: quantification of the PI-stained migrated cells. Mean numbers of cells from three independent experiments are shown with SD error bars. **P < 0.01, ***P < 0.005 (Student’s t-test) vs. siCtl. Bottom panel: western blot showing expression of YTHDF1 in control (siCtl) and YTHDF1-targeting siRNA (siYTHDF1#1, siYTHDF1#2) transfected cells subjected to transwell migration assay. GAPDH used as a loading control

The presence of a subpopulation of stem cell-like cells in tumors, known as cancer stem cells (CSCs), is known to be the major factor of cancer recurrence and metastatic potential. Therefore, we sought to investigate the effect of YTHDF1 on CSC properties of GBM. DBTRG-05MG cells were transduced with two lentivirus constructs encoding YTHDF1-targeting shRNAs, and sphere-formation assay was performed to assess the presence of CSCs. Whereas the cells transduced with empty vector control (pLKO.1) could efficiently form the tumorspheres, the cells transduced with shRNA-encoding lentiviruses had significantly reduced sphere-forming capacity (Fig. 3a). As was shown by immunoblotting, the levels of CSC markers, CD133, NANOG, OCT4 and REX1, were markedly reduced upon YTHDF1 knockdown (Fig. 3b). Of note, the expression of SOX2 appeared to fluctuate, which could be a cell line-specific phenomenon (Fig. 3b). Since CSCs are characterized by increased migration and metastatic capacity, we performed transwell migration assay on DBTRG-05MG cells transfected with YTHDF1-targeting siRNAs. The knockdown of YTHDF1 resulted in significantly reduced cell migration through the transwells (Fig. 3c, d), which correlated with the reduced stem cell properties of GBM cell population.

### MSI1 positively regulates the expression of YTHDF1 through stabilization of mRNA in a GBM cell line

**Fig. 4:**
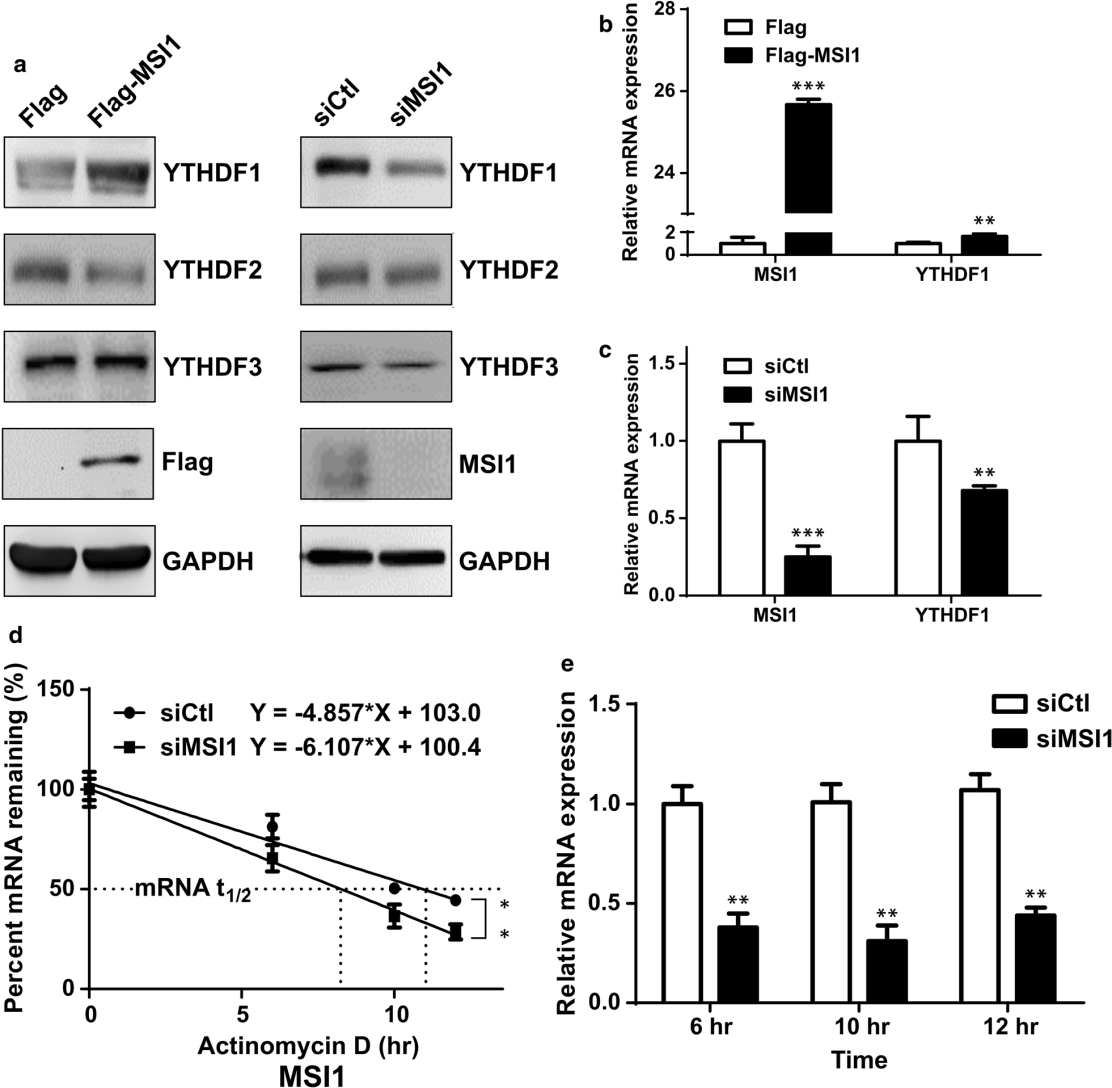
MSI1 positively regulates the expression of YTHDF1 through stabilization of ***YTHDF1*** mRNA in GBM cell line. **a** Western blot analysis of the expression of different YTH domain family proteins in DBTRG-05MG cells transfected with FLAG-tagged MSI1 (left panel) or MSI1-targeting siRNA (right panel). GAPDH used as a loading control. **b** qRT-PCR analysis of *MSI1* and *YTHDF1* mRNA levels in DBTRG-05MG cells transfected with FLAG-tagged MSI1. Data are expressed relative to cells transfected with empty vector (Flag). Means from three separate experiments are shown with SD error bars. **P < 0.01, ***P < 0.005 (Student’s t-test). **c** qRT-PCR analysis of *MSI1* and *YTHDF1* mRNA levels in DBTRG-05MG cells transfected with MSI1-targeting siRNA. Data are expressed relative to cells transfected with scrambled sirNA control (siCtl). Means from three separate experiments are shown with SD error bars. **P < 0.01, ***P < 0.005 (Student’s t-test). **d** Assay showing stability of *YTHDF1* mRNA in DBTRG-05MG cells transfected MSI1-targeting siRNA (siMSI1) as compared to scrambled siRNA control (siCtl). Transcription was blocked by treatment of cells with actinomycin D and *YTHDF1* mRNA levels were assessed by qRT-PCT at the indicated time points. Data are expressed relative to the siCtl control. Means from three separate experiments are shown with SD error bars. **P < 0.01, ***P < 0.005 (Student’s t-test). **e** The knockdown efficiency of *MSI1* mRNA assessed by qRT-PCR. Quantitative data are presented as means from three independent experiments with SD error bars. **P < 0.01 (Student’s t-test)

Previously, we demonstrated that RNA-binding protein Musashi-1 (MSI1) plays an important tumorigenic role in GBM, controlling such processes as cell migration and drug resistance [25–27]. Given such functional overlap between MSI1 and YTHDF1, we investigated the possibility of these two proteins to be involved in the same pathway. Here, we show that overexpression of MSI1 leads to upregulation of YTHDF1 protein levels, and conversely, siRNA-mediated knockdown of MSI1 results in YTHDF1 downregulation (Fig. 4a). This effect was specific to YTHDF1 only, but not to other members of this family, YTHDF2 and YTHDF3 (Fig. 4a). As was shown by qRT-PCR, MSI1 overexpression led to upregulation of *YTHDF1* mRNA level (Fig. 4b), and MSI1 knockdown led to *YTHDF1* mRNA downregulation (Fig. 4c). Since MSI1 is an RNA-binding protein that regulates gene expression post-transcriptionally, its positive effect on *YTHDF1* mRNA could be due to its stabilization. Therefore, we used actinomycin D to block transcription and monitored the stability of *YTHDF1* mRNA in a time course of 10 hours in DBTRG-05MG cells transfected with MSI1-targeting siRNA (Fig. 4d, e). It was shown that the knockdown of MSI1 led to markedly decreased stability of *YTHDF1* mRNA (Fig. 4d).

### YTHDF1 mediates MSI1-dependent enhancement of cell proliferation

**Fig. 5:**
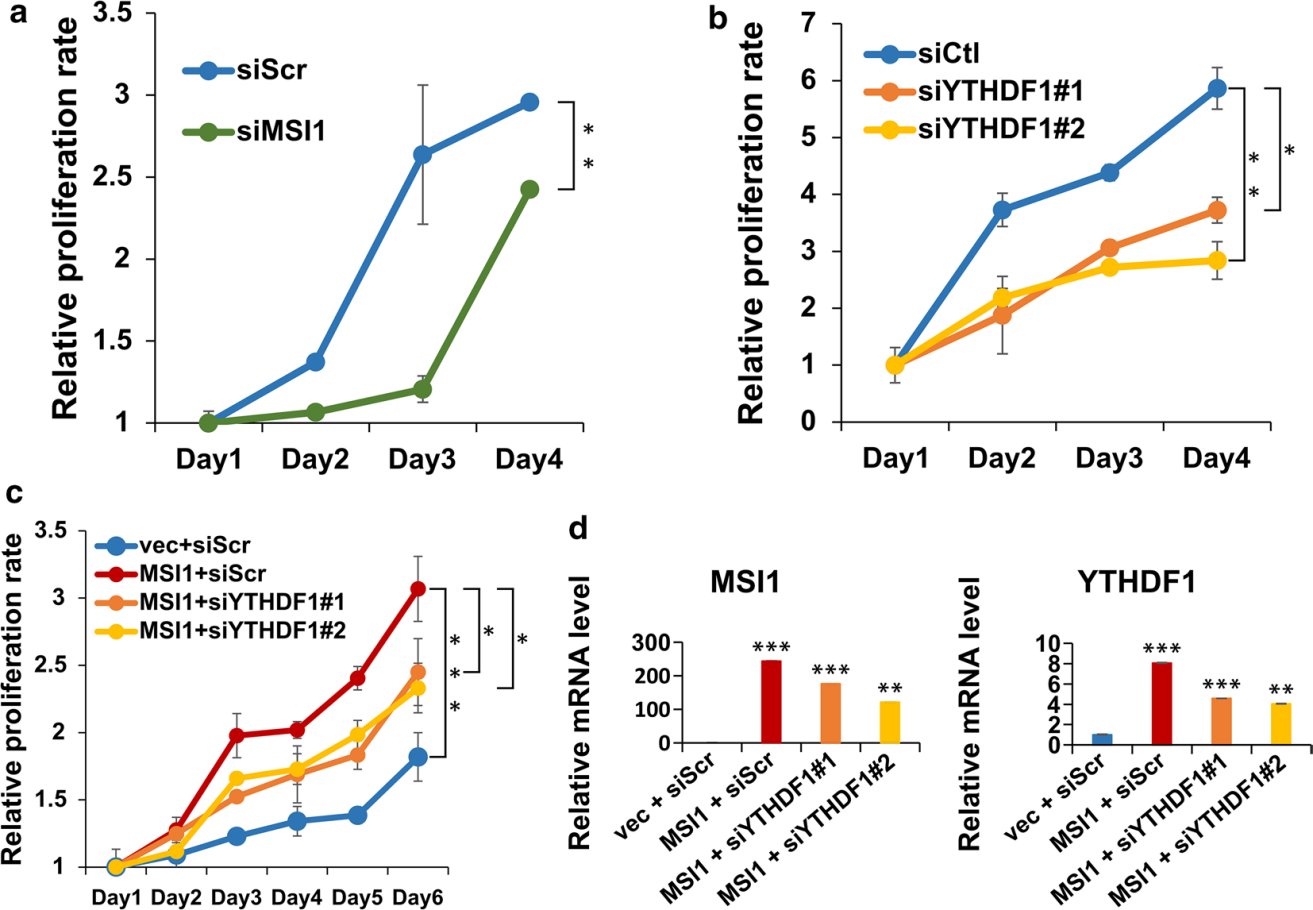
YTHDF1 mediates MSI1-dependent enhancement of cell proliferation.** a** Cell proliferation assay performed on DBTRG-05MG cells transfected with scrambled control (siScr) and MSI1-targeting siRNA (siMSI1). **b** Cell proliferation assay performed on DBTRG-05MG cells transfected with control siRNA (siCtl) and two YTHDF1-targeting siRNAs (siYTHDF1#1 and siYTHDF1#2). **c** Cell proliferation assay performed on DBTRG-05MG cells transfected with the indicated combinations of MSI1-overexpressing plasmid (MSI1) and YTHDF1-targeting siRNAs (siYTHDF1#1 and siYTHDF1#2). siScr – siRNA control, vec – overexpression plasmid control. The proliferation rate in **a**, **b**, and **c** was calculated as the mean ratio of MTT absorbance on the indicated days to that on day 1. Means from three separate experiments are shown with SD error bars. **d** qRT-PCR validation of overexpression of *MSI1* (left panel) and knockdown of *YTHDF1* (right panel) in the experiment shown in **c**. All data are rpesented as means from three separate experiments with SD error bars. *P < 0.05, **P < 0.01, ***P < 0.005 (Student’s t-test)

After demonstrating that MSI1 directly regulated YTHDF1 expression by stabilizing its mRNA, we sought to investigate the functional implications of MSI1-YTHDF1 pathway. Importantly, the knockdown of both MSI1 (Fig. 5a) and YTHDF1 (Fig. 5b) led to decreased proliferation of DBTRG-05MG cells. Conversely, overexpression of MSI1 resulted in increased proliferation of DBTRG-05MG cells (Fig. 5c, d). However, this pro-proliferative effect of MSI1 was significantly reduced when MSI1-encoding plasmid was cotransfected with siRNAs targeting YTHDF1 (Fig. 5c, d). Thus, we can conclude that MSI1 enhances proliferation of GBM cells by positively regulating YTHDF expression.

### YTHDF1 mediates MSI1-dependent enhancement of cell mobility

**Fig. 6:**
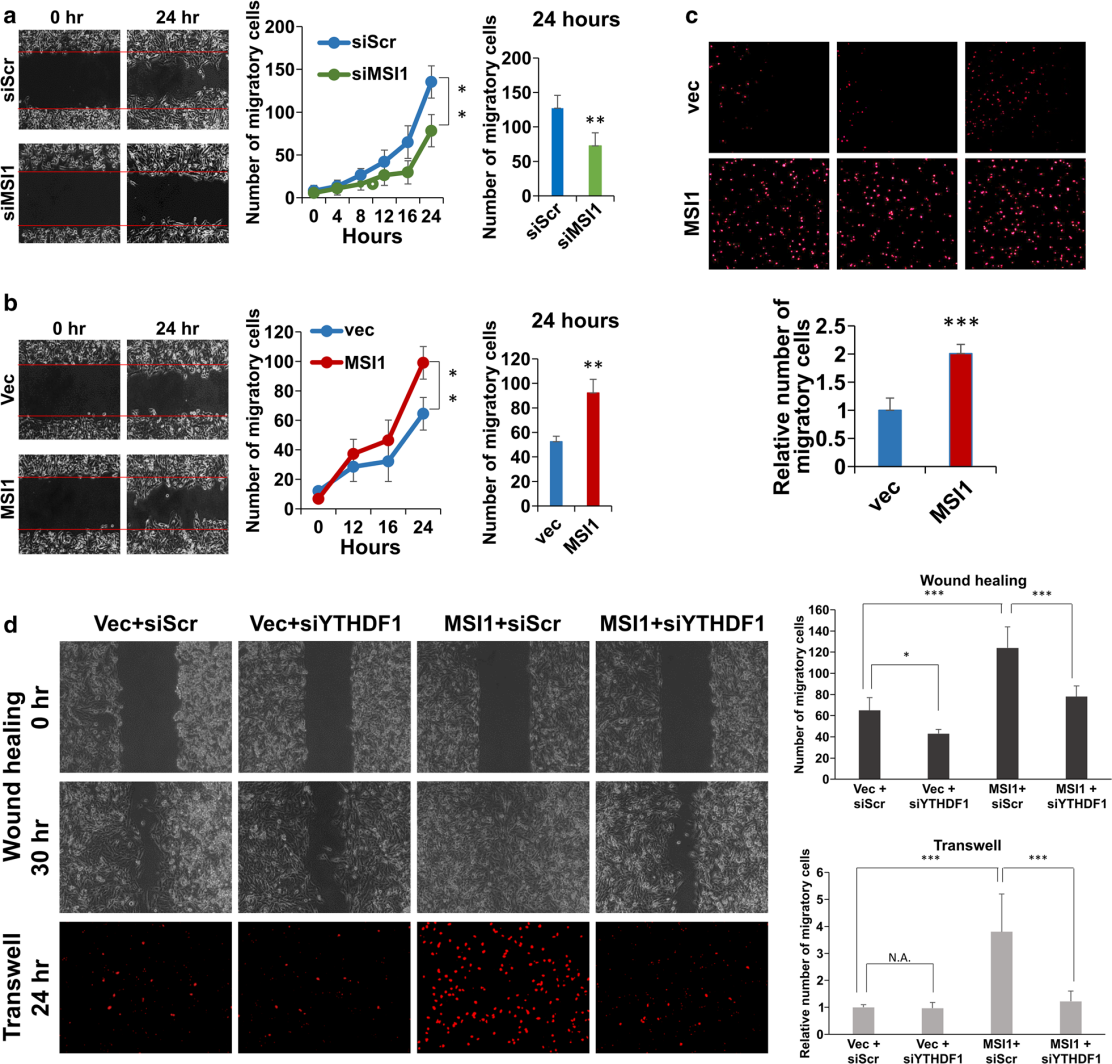
YTHDF1 mediates MSI1-dependent enhancement of cell mobility.** a** Wound healing assay performed on DBTRG-05MG cells transfected with siRNA scrambled control (siScr) and MSI1-targeting siRNA (siMSI1). **b** Wound healing assay performed on DBTRG-05MG cells transfected with siRNA scrambled control (siScr) and MSI1-targeting siRNA (siMSI1). In **a** and **b**, left panel: images of wounds at the beginning (0 hr) and end (24 hr) of the assay; middle panel: quantification of migratory cells in a time course of 24 h; right panel: quantification of migratory cells at the end point of the assay (24 h). **c** Transwell migration assay performed on DBTRG-05MG cells transfected with empty vector control (vec) and MSI1-encoding plasmid (MSI1). Top panel: fluorescent images of migrated cells stained with propidium iodide (triplicate). Bottom panel: quantification of migrated cells. **d** Wound healing (top two panels) and transwell migration (bottom panel) assays performed on DBTRG-05MG cells transfected with the indicated combinations of siRNAs (siScr and siYTHDF1) and plasmids (vec and MSI1). Right panel: quantification of the assays. All quantifiable data are presented as the means from three biological replicates with SD error bars. *P < 0.05, **P < 0.01, ***P < 0.005 (Student’s t-test)

Given that the cell migration capacity of cancer cells that ensures the metastasis is one of the hallmarks of cancer [28], we applied the wound healing assay and identified that the knockdown of MSI1 could reduce the migration of DBTRG-05MG cells (Fig. 6a), whereas its overexpression, on the contrary, increased the migration capacity (Fig. 6b). The positive effect of MSI1 overexpression on cell migration was also confirmed by transwell migration assay (Fig. 6c). However, when MSI1 was overexpressed together with YTHDF1-targeting siRNA, the positive effect on cell migration was substantially reduced as was demonstrated by wound healing and transwell migration assays (Fig. 6d). Thus, we could conclude that MSI1 enhanced cell migration of GBM cells by positively regulating YTHDF expression.

### MSI1 and YTHDF1 are associated with lower survival of glioma patients

**Fig. 7:**
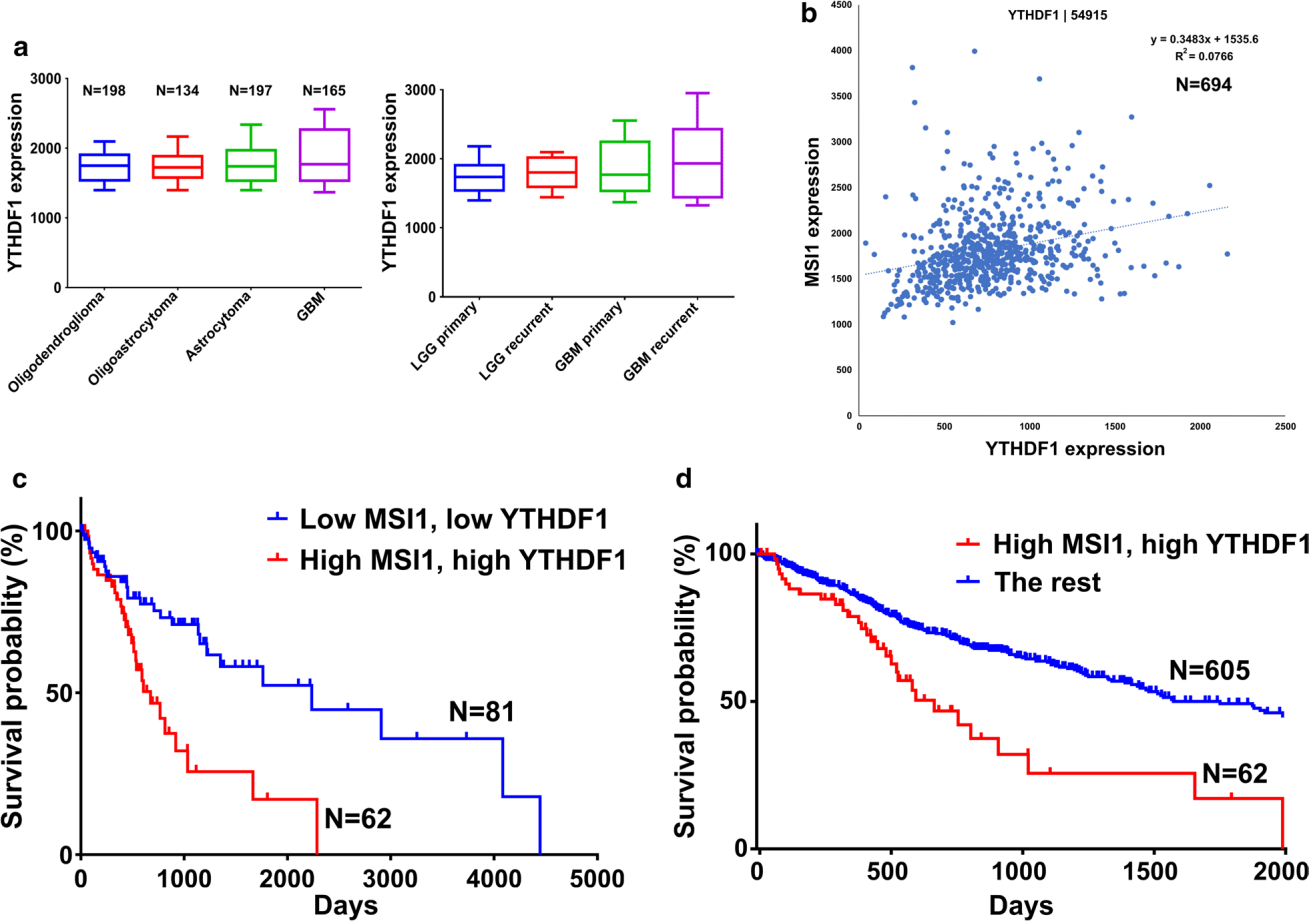
MSI1 and YTHDF1 are associated with lower survival of glioma patients.** a** Box plots showing the expression of YTHDF1 in three stages of low grade glioma (LGG) and GBM (left panel); in primary and recurrent LGG and GBM (right panel). **b** Scatter plot showing positive correlation of expression of *MSI1* and *YTHDF1* in the glioma dataset. **c** Kaplan-Meier plot showing survival analysis in the groups of glioma patients with low expression of *MSI1* and *YTHDF1* (below the lower quartile) and high expression of *MSI1* and *YTHDF1* (above the higher quartile). **d** Kaplan-Meier plot showing survival analysis in the group of patients with high expression of *MSI1* and *YTHDF1* (above the higher quartile), and the rest of patients (expression of *MSI1* and *YTHDF1* below the higher quartile)

In light of the pro-oncogenic role of MSI1/YTHDF1 pathway revealed in a GBM cell line, we proceeded to investigate the biomarker potential of these proteins in clinical settings. For this purpose, we used the RNA-Seq glioma dataset from the TCGA database, which included the samples from both GBM and low grade gliomas (LGGs), such as oligodendroglioma, oligoastrocytoma, and astrocytoma. The expression of *YTHDF1* was proportional to the brain tumor grade with the statistical significance of GBM vs. oligodendroglioma *p* = 0.0001, GBM vs. oligoastrocytoma *p* = 0.001, and GBM vs. astrocytoma *p* = 0.04 (Fig. 7a, left panel). The recurrent tumors were characterized by a trend of higher expression of *YTHDF1* compared to the primary tumors (Fig. 7a, right panel). *MSI1* and *YTHDF1* demonstrated mildly positive correlation of expression in glioma datasets (Fig. 7b). To identify the biomarker potential of *MSI1* and *YTHDF1* expression, the survival analysis was performed. The survival of two groups of patients with GBM and LGGs was estimated: a group of 62 patients with high expression of both *MSI1* and *YTHDF1* (expression levels above the upper quartile), and a group of 81 patients with low expression of both *MSI1* and *YTHDF1* (expression levels below the lower quartile) (Fig. 7c). The latter group demonstrated significantly better survival rate with a hazard ratio of 0.3529. Similarly, when the survival of the group with high expression of both *MSI1* and *YTHDF1* was compared with the rest of patients in the dataset (expression levels of *MSI1* and *YTHDF1* below the upper quartile), the former group was shown to have poor survival with a hazard ratio of 3.126 (Fig. 7d). To summarize, *MSI1* and *YTHDF1* can be considered as negative prognostic markers in gliomas.

## Discussion

Whereas the role of epigenetic modifications of DNA and histones in promoting pathological features of GBM such as chemoresistance, recurrence and invasiveness was widely investigated, relatively little is known about the role of m6A epitranscriptomic modifications in GBM tumor progression. Given that the functional effects of m6A methylation are dependent on the m6A reader proteins of the YTH domain family, we screened the Oncomine database to study the expression of five members of this family in multiple GBM datasets, and found that one of them, YTHDF1, was the most highly upregulated in GBM as compared to the normal tissues (Fig. 1a). Moreover, YTHDF1 was the most specifically expressed in brain and CNS cancers comparing to other types of tumors (Fig. 1b). The aberrant expression and pro-oncogenic role of YTHDF1 have been demonstrated for several types of cancer such as hepatocellular carcinoma [29], colorectal cancer [30], lung cancer [21]. To our knowledge, this is the first study demonstrating the pro-oncogenic role of YTHDF1 in GBM.

To characterize the functional role of YTHDF1 in GBM, we performed its knockdown in a GBM cell line. We observed that the ablation of YTHDF1 led to decreased proliferation of GBM cells (Fig. 2c), and secondly, sensitized them to TMZ, the most common anti-GBM chemotherapy drug (Fig. 2d). Consistently with our results, YTHDF1 ablation has previously been shown to result in decreased proliferation in different types of cancer, including colorectal [30] and lung [21] carcinomas. As was previously shown by proteomic analysis of lung carcinoma cells, the knockdown of YTHDF1 led to activation of cell cycle inhibitor p27^Kip1^ and suppression of cell cycle activator genes encoding CDK2, CDK4 and cyclin D1 [21].

It is widely believed that due to epigenetic alterations, the dynamic equilibrium exists between CSCs and differentiated GBM cells, as different stimuli can cause differentiation of CSCs to non-CSCs and reverse dedifferentiation of non-CSCs to CSCs [31]. Therefore, we tested the possibility of epitranscriptome regulator, YTHDF1, to be involved in stemness properties of GBM. We demonstrate that the knockdown of YTHDF1 expression leads to decreased tumorsphere formation (Fig. 3a), decreased expression of stemness markers (Fig. 3b), and reduced migration capacity (Fig. 3c), all these observations are indicative of the reduction of a subpopulation of CSCs in a GBM cell line. Since GBM CSCs are characterized by increased resistance to TMZ [32], YTHDF1 knockdown-mediated reduction of CSCs may explain the elevated sensitivity of GBM cells to this drug (Fig. 2d). In carcinomas, such as hepatocellular carcinoma, YTHDF1 was shown to increase the expression of SNAIL, which is the master regulator of epithelial-mesenchymal transition program associated with increased cell invasion [33].

MSI1 is an RBP that is overexpressed in GBM and serves as an unfavorable prognostic biomarker [5, 6]. Importantly, MSI1 is a well-known factor in the maintenance of stem cells in various tissues [8]. Consistently with its high expression in cancers, and its importance in stem cell signaling, MSI1 has been implicated in CSC properties of different tumors, including GBM [9, 10]. In this study, we found that YTHDF1 is positively regulated by MSI1, as was demonstrated by MSI1 overexpression (Fig. 4a) and knockdown (Fig. 4c). Initially, MSI1 was identified as a negative regulator of translation of mRNAs. For example, MSI1 negatively regulates the translation of its most well-characterized target, *NUMB1* mRNA, by binding to its 3’-UTR and interacting with poly(A)-binding protein (PABP), thus preventing its function in cap-dependent initiation of translation [34]. Similarly, MSI1 was shown to suppress the translation of CDKN1A gene encoding cell cycle inhibitor p21^Cip1^ [35]. At the same time, depending on the context, MSI1 can serve as a positive regulator of translation, which may also involve the mechanism of interaction with PABP [12]. Here, we demonstrate that MSI1 increases the stability of YTHDF1 mRNA, which may contribute to the mechanism of MSI-dependent upregulation of YTHDF1 protein expression (Fig. 4d). Previously, MSI1-mediated stabilization of mRNAs was shown to occur by such mechanisms as competing with miRNA-binding sites [36], or controlling the length of poly(A) tail by recruiting GLD2 poly(A) polymerase [37]. Since we observed only marginal effect of MSI1 knockdown on *YTHDF1* mRNA stability (Fig. 4d), we believe that translational regulation may still serve as the principal mechanism of positive control of YTHDF1 expression.

Here, we found that the inhibitory effects imposed on the processes of proliferation and migration by YTHDF1 knockdown, were partially rescued by the concomitant overexpression of MSI1 (Figs. 5 and 6). In previous studies, several mechanisms of regulation of cell migration by MSI1 in GBM were delineated. MSI1 was shown to be a direct translational activator of ICAM1, a cell surface protein directly involved in cancer cell migration [38]. In the study by Chen et al., it was shown that MSI1 enhanced GBM cell migration by inhibiting the expression of tensin-3, the cytoskeleton protein that inhibits cell motility [26]. Currently, there is no mechanistic understanding of how YTHDF1 regulates these processes in GBM, however, given the fact that MSI1 partially rescues the suppression of proliferation and cell migration induced by YTHDF1 depletion, there may be an overlap between the pathways regulated by these two proteins.

**Fig. 8:**
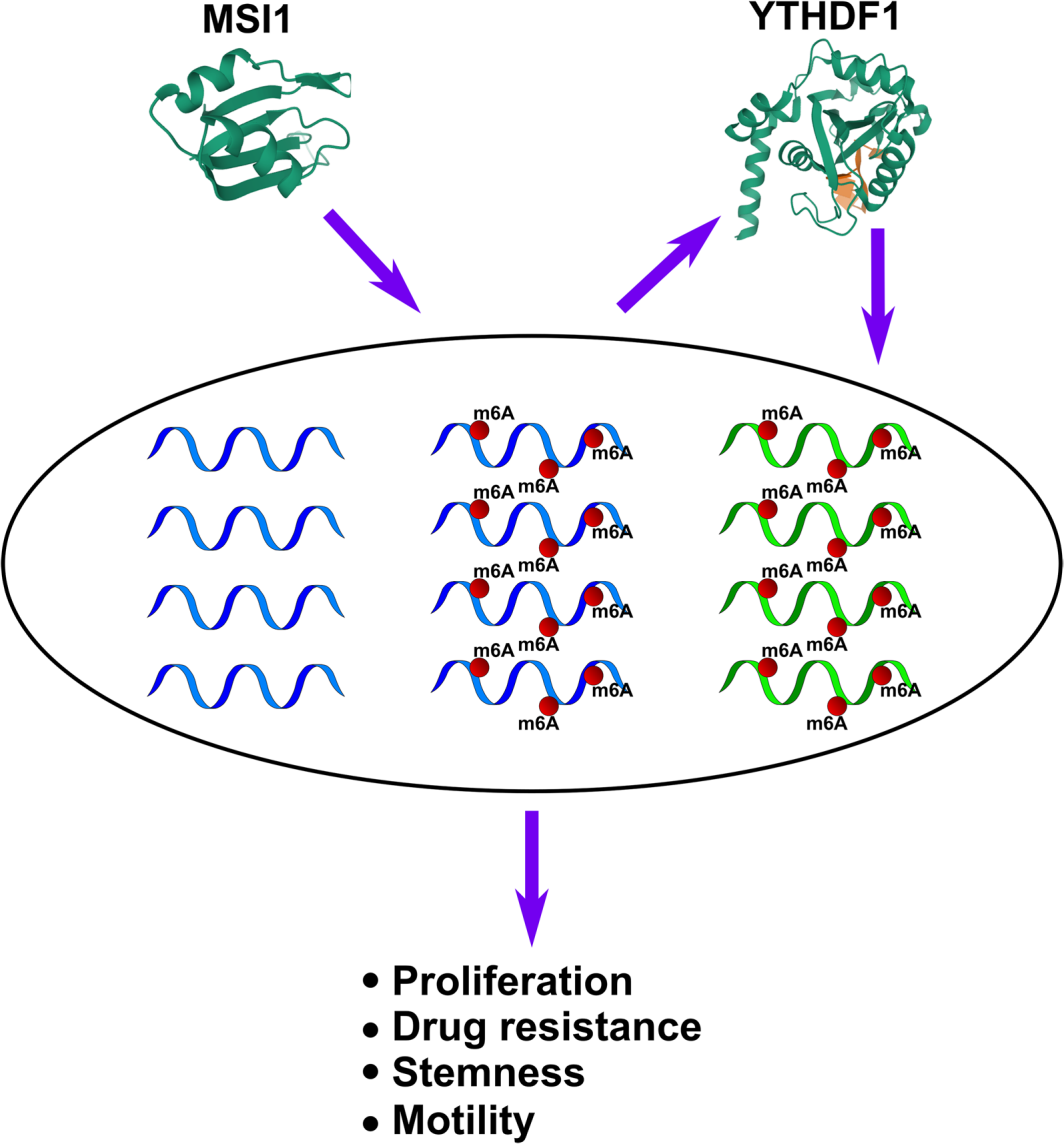
Summary of the pathways regulated by MSI1 and YTHDF1. Both proteins are global mRNA regulators that may have unique (blue RNAs without red balls for MSI1 and green RNAs with red balls for YTHDF1) and common (blue RNAs with red balls) mRNA targets, which results in enhancement of such pro-oncogenic properties as proliferation, drug resistance, stemness and motility. At the same, MSI1-mediated regulation of transcriptome results in upregulation of YTHDF1

As a conclusion, in this study, for the first time we show that YTHDF1 regulates such pro-oncogenic features of GBM as increased proliferation, drug resistance, cell migration, and tumorigenic efficiency determined by tumorsphere formation assay, the properties which are commonly attributed to CSCs. MSI1, the protein that was previously characterized to be highly unfavorable pro-oncogenic factor in GBM and a regulator of stem cell state, was shown here to upregulate YTHDF1 and partially recover the effects of YTHDF1 knockdown. Both YTHDF1 and MSI1 are master posttranscriptional regulators that globally alter gene expression. Therefore, these two proteins may regulate a number of unique and overlapping pathways that lead to increased GBM malignancy and cancer stemness properties, but these pathways are intimately connected by the direct regulation of YTHDF1 by MSI1 (Fig. 8). We propose that any future studies aimed to uncover the prognostic value and therapy target potential of these two genes, should consider them together.

## Conclusions

We demonstrated that YTHDF1 is involved in MSI1-mediated GBM tumorigenesis processes such as cell proliferation and migration, and also regulate the stem-like properties of GBM cells. We also identified the direct regulation of YTHDF1 by MSI1. The concomitant upregulation of MSI1 and YTHDF1 was associated with decreased survival of glioma patients. MSI1 and YTHDF1 can be considered as negative prognostic markers in gliomas.

## Data Availability

The datasets used and/or analyzed during the current study are available from the corresponding author on reasonable request.

## Abbreviations

GBM: Glioblastoma
LGG: Low grade glioma
TMZ: Temozolomide
CSC: Cancer stem cell
RBP: RNA-binding protein.
